# Exploratory subgroup analysis of patients with prior trastuzumab use in the ATTRACTION-2 trial: a randomized phase III clinical trial investigating the efficacy and safety of nivolumab in patients with advanced gastric/gastroesophageal junction cancer

**DOI:** 10.1007/s10120-019-00970-8

**Published:** 2019-05-13

**Authors:** Taroh Satoh, Yoon-Koo Kang, Yee Chao, Min-Hee Ryu, Ken Kato, Hyun Cheol Chung, Jen-Shi Chen, Kei Muro, Won Ki Kang, Kun-Huei Yeh, Takaki Yoshikawa, Sang Cheul Oh, Li-Yuan Bai, Takao Tamura, Keun-Wook Lee, Yasuo Hamamoto, Jong Gwang Kim, Keisho Chin, Do-Youn Oh, Keiko Minashi, Jae Yong Cho, Masahiro Tsuda, Mitsunobu Tanimoto, Li-Tzong Chen, Narikazu Boku

**Affiliations:** 1 Frontier Science for Cancer and Chemotherapy, Osaka University Graduate School of Medicine, Suita, Japan; 2 Department of Oncology, University of Ulsan College of Medicine, Asan Medical Center, Seoul, South Korea; 3 Department of Oncology, Taipei Veterans General Hospital, Taipei, Taiwan; 4 Division of Gastrointestinal Medical Oncology, National Cancer Center Hospital, 5-1-1, Tsukiji, Chuo-ku, Tokyo, 104-0045 Japan; 5 Division of Medical Oncology, Yonsei Cancer Center, Song-Dang Institute for Cancer Research, Yonsei University College of Medicine, Yonsei University Health System, Seoul, South Korea; 6 Division of Hematology and Oncology, Department of Internal Medicine, Linkou Chang Gung Memorial Hospital, Chang Gung University, Taoyuan, Taiwan; 7 Department of Clinical Oncology, Aichi Cancer Center Hospital, Nagoya, Japan; 8 Division of Hematology-Oncology, Department of Medicine, Samsung Medical Center, Sungkyunkwan University School of Medicine, Seoul, South Korea; 9 Department of Oncology, National Taiwan University Hospital, and National Taiwan University Cancer Center, Taipei, Taiwan; 10 Department of Gastrointestinal Surgery, Kanagawa Cancer Center, Yokohama, Japan; 11 Present Address: Department of Gastric Surgery, National Cancer Center Hospital, Tokyo, Japan; 12 Division of Hematology and Oncology, Department of Internal Medicine, Korea University College of Medicine, Seoul, South Korea; 13 Division of Hematology and Oncology, Department of Internal Medicine, China Medical University Hospital, China Medical University, Taichung, Taiwan; 14 Department of Medical Oncology, Faculty of Medicine, Kindai University, Osaka, Japan; 15 Present Address: Department of Medical Oncology, Kindai University Nara Hospital, Ikoma, Japan; 16 Division of Hematology and Oncology, Department of Internal Medicine, Seoul National University Bundang Hospital, Seoul National University College of Medicine, Seongnam, South Korea; 17 Keio Cancer Center, Keio University School of Medicine, Tokyo, Japan; 18 School of Medicine, Kyungpook National University, Daegu, South Korea; 19 Department of Gastroenterology, Cancer Institute Hospital of the Japanese Foundation for Cancer Research, Tokyo, Japan; 20 Department of Internal Medicine, Seoul National University Hospital, Cancer Research Institute, Seoul National University College of Medicine, Seoul, South Korea; 21 Clinical Trial Promotion Department, Chiba Cancer Center, Chiba, Japan; 22 Department of Medical Oncology, Gangnam Severance Hospital, Yonsei University College of Medicine, Seoul, South Korea; 23 Department of Gastroenterological Oncology, Hyogo Cancer Center, Akashi, Japan; 24 Oncology Clinical Development Planning, Oncology Clinical Development Unit, ONO Pharmaceutical Co., Ltd., Osaka, Japan; 25 National Institute of Cancer Research, National Health Research Institutes, and National Cheng Kung University Hospital, National Cheng Kung University, Tainan, Taiwan

**Keywords:** Nivolumab, Gastric cancer, Gastroesophageal junction cancer, Trastuzumab

## Abstract

**Background:**

Data on immune checkpoint inhibitor efficacy in patients with human epidermal growth factor receptor 2-positive (HER2+) advanced gastric/gastroesophageal junction (G/GEJ) cancer are lacking. Because HER2 status was not captured in the ATTRACTION-2 trial, we used patients with prior trastuzumab use (Tmab+) as surrogate for HER2 expression status to evaluate the efficacy and safety of nivolumab as third- or later-line therapy in these patients.

**Methods:**

In ATTRACTION-2, a randomized, double-blind, placebo-controlled, phase 3 multicenter trial, patients were randomized (2:1) to receive nivolumab (3 mg/kg) or placebo every 2 weeks until disease progression or toxicity requiring study discontinuation. Overall survival (OS), progression-free survival (PFS), objective response rate (ORR), and safety were assessed.

**Results:**

Of 493 enrolled patients, 81 (nivolumab, *n* = 59; placebo, *n* = 22) were Tmab+ and 412 (nivolumab, *n* = 271; placebo, *n* = 141) were Tmab−. In both groups, patients receiving nivolumab showed a longer median OS vs placebo (Tmab+, 8.3 [95% confidence interval, 5.3–12.9] vs 3.1 [1.9–5.3] months, hazard ratio, 0.38 [0.22–0.66]; *P* = 0.0006; Tmab−, 4.8 [4.1–6.0] vs 4.2 [3.6–4.9] months, 0.71 [0.57–0.88]; *P* = 0.0022). PFS was longer in both groups receiving nivolumab vs placebo (Tmab+, 1.6 [1.5–4.0] vs 1.5 [1.3–2.9] months, 0.49 [0.29–0.85]; *P* = 0.0111; Tmab−, 1.6 [1.5–2.4] vs 1.5 [1.5–1.5] months, 0.64 [0.51–0.80]; *P* = 0.0001).

**Conclusions:**

Nivolumab was efficacious and safe as third- or later-line therapy regardless of prior trastuzumab use in patients with advanced G/GEJ cancer.

## Introduction

Gastric/gastroesophageal junction (G/GEJ) cancer is the fifth most common cancer and the third leading cause of cancer-related deaths globally, according to the World Health Organization’s latest estimates [1]. Nearly 1 million new cases and ~ 725,000 deaths attributable to G/GEJ cancer occurred in 2012, with the highest incidence and mortality rates reported in East Asia [1].

First-line standard of care in patients with human epidermal growth factor receptor 2-negative (HER2−), unresectable, or recurrent G/GEJ cancer includes a two-drug combination of platinum-based agents and fluoropyrimidines, e.g., cisplatin, oxaliplatin, S-1, capecitabine, or 5-fluorouracil. In some medically fit patients with good performance status, a third drug, e.g., docetaxel or epirubicin, may be added [2-4]. Depending on the method of assessment, ~ 6 to ~ 37% of all G/GEJ cancers show HER2 overexpression [5, 6]. Based on the results of the ToGA study [7], trastuzumab is now recommended in combination with chemotherapy as first-line therapy in patients with HER2-positive (HER2+) metastatic or advanced G/GEJ cancer [2-4].

While trastuzumab showed a survival benefit as first-line chemotherapy for HER2+ G/GEJ cancer patients in the ToGA study, none of the other anti-HER2 drugs, such as lapatinib or trastuzumab–emtansine (T-DM1), or continuing trastuzumab beyond progression in second-line treatment showed benefits [8-10]. Recently, studies evaluating trastuzumab resistance mechanisms have highlighted certain pathways for resistance. In the multicenter, prospective, case–control AMNESIA study in patients with HER2+ metastatic gastric cancer, genomic alterations in the epidermal growth factor receptor (EGFR)/MET/KRAS/PI3K/PTEN pathway were significantly more frequent in trastuzumab-resistant (55%) patients than in trastuzumab-sensitive (0%) patients, and patients without these genomic alterations had a significantly longer median progression-free survival (PFS) (5.2 vs 2.6 months, hazard ratio [HR], 0.34 [95% confidence interval (CI), 0.07–0.48]; *P* = 0.001) and overall survival (OS) (16.1 vs 7.6 months; HR, 0.38 [95% CI 0.09–0.75]; *P* = 0.015) than those with alterations [11]. At present, however, no chemotherapy regimen specific to HER2+ G/GEJ cancer patients has been established in the second- or later-line treatment setting.

In the ATTRACTION-2 trial [12], nivolumab, an anti-programmed death-1 (PD-1) immunoglobulin G4 antibody, demonstrated significant survival benefits compared with placebo as the salvage-line treatment for patients with advanced G/GEJ cancer who were previously treated with ≥ 2 chemotherapy regimens (median OS: nivolumab, 5.26 months [95% CI 4.60–6.37]; placebo, 4.14 months [3.42–4.86]; HR 0.63 [0.51–0.78]; *P* < 0.0001). In Japan, nivolumab monotherapy is recommended as third-line treatment after the results of the ATTRACTION-2 study demonstrated its efficacy [13], whereas in the United States, pembrolizumab is recommended for third- or later-line treatment in patients with PD-ligand 1 (PD-L1+)  advanced G/GEJ cancer based on findings from the KEYNOTE-059 study [14] regardless of prior chemotherapy regimens.

The relationship between HER2 and PD-L1 expression has been assessed in a few studies recently; however, there are conflicting conclusions regarding their correlation [15-18]. In a study evaluating the expression and impact of PD-L1/PD-1 in gastric cancer in Caucasian patients, almost 50% of PD-L1+ patients were HER2+ [15]. In in vitro studies in HER2-overexpressing cell lines, PD-L1 expression decreased in a dose- and time-dependent manner after EGFR/HER2-targeted treatment [17]. In contrast, in a retrospective analysis of resected, treatment-naïve gastric cancers, PD-L1 expression was observed more frequently in the HER2 − group than in the HER2+ group (39.0% vs 24.2%; *P* = 0.020) [18]. Thus, discrepancies concerning the relationship between HER2 and PD-L1 expression exist and data related to the combined or sequential use of anti-HER2 and anti–PD-1/PD-L1 agents are also lacking. In the ATTRACTION-2 trial, we did not observe a clear relationship between PD-L1 expression and efficacy of nivolumab; furthermore, there is no evidence in literature regarding the outcome of immune checkpoint inhibitor treatment, including nivolumab, on HER2+ patients. Although HER2 status was not captured in ATTRACTION-2, the study did include patients who had previously used or not used trastuzumab. Therefore, exploratory analysis of ATTRACTION-2 data based on prior trastuzumab-use status could serve as surrogate for HER2 expression status.

The objective of this post hoc analysis of the ATTRACTION-2 trial was to assess the efficacy and safety of nivolumab for advanced G/GEJ cancer in patients who had previously used trastuzumab.

## Methods

### Study design

In this post hoc analysis of the ATTRACTION-2 trial [12], a randomized, double-blind, placebo-controlled, phase 3 trial conducted at 49 sites in Japan, South Korea, and Taiwan between October 2014 and August 2016, the efficacy and safety of nivolumab were studied according to previous use of trastuzumab. ATTRACTION-2 (ClinicalTrials.gov ID: NCT02267343) was conducted in accordance with the Declaration of Helsinki and the Good Clinical Practice guidelines developed by the International Conference on Harmonisation of Technical Requirements for Registration of Pharmaceuticals for Human Use [19]. The protocol was approved by the institutional review board or independent ethics committee for each study center. Written informed consent was obtained from all patients.

### Patients

Patients were eligible if they were aged ≥ 20 years, had unresectable or metastatic, histologically confirmed G/GEJ adenocarcinoma, were treated with ≥ 2 previous chemotherapy regimens, were refractory to or intolerant of standard therapy in the advanced or recurrent setting, had not planned to receive new chemotherapy including antibodies, had an Eastern Cooperative Oncology Group performance status of 0 or 1, and had a life expectancy of ≥ 3 months. Patients were excluded if they had an ongoing or previous autoimmune or interstitial lung disease; active diverticulitis or gastrointestinal ulcerative disease, or other uncontrolled or clinically significant medical disorder; brain metastases that were symptomatic or required treatment; and had previously been treated with anti-PD-1, anti-PD-L1 or anti-PD-L2, anti-CD137, or anti-CTLA-4 antibodies.

### Treatment

Enrolled patients were randomized in a 2:1 ratio via an interactive web response system to receive nivolumab or placebo. Patients received 3 mg/kg nivolumab or placebo intravenously every 2 weeks for 6 weeks (one treatment cycle). Dose modification was allowed in case of a ≥ 10% change in body weight after randomization. Study treatment was continued until progressive disease (PD) evaluated by an investigator or onset of toxicity requiring permanent discontinuation of study treatment. Patients could continue study treatment after the first episode of PD if they showed evidence of investigator-assessed clinical benefit, tolerance of study drug, and stable performance status; if continuation of treatment would not delay an intervention to prevent serious complications of disease progression; and if the patient provided written informed consent before continuing the study treatment.

### Endpoints

The primary endpoint was OS. Secondary efficacy endpoints included PFS, objective response rate (ORR; proportion of patients with confirmed complete response [CR] or partial response [PR]), disease control rate (DCR; proportion of patients with confirmed CR, PR, or stable disease), duration of response (DOR), time to response (TTR), best overall response (BOR), and maximum percentage change from baseline in the sum of diameters of target lesions. Safety endpoints included adverse events (AEs) and treatment-related AEs occurring through the study period.

### Assessments

Tumor responses were assessed via computed tomography or magnetic resonance imaging according to Response Evaluation Criteria In Solid Tumors guidelines version 1.1. Tumor assessment was repeated after each cycle for ten cycles ( ~ 14 months), then after every two treatment cycles until discontinuation of study treatment because of PD, initiation of post-study treatment, or assessment of PD in patients who discontinued because of toxicity. Tumor assessments were also performed at the end of treatment. AEs were evaluated according to the National Cancer Institute Common Terminology Criteria for Adverse Events version 4.0 continuously during treatment and for 28 days thereafter. For patients with available tumor samples, PD-L1 tumor expression was determined retrospectively by immunohistochemistry performed at a central laboratory (28-8 pharmDx assay; Dako, Carpinteria, CA, USA). PD-L1 positivity was defined as staining in ≥ 1% of tumor cells.

### Statistical analysis

A subgroup analysis by history of trastuzumab use was conducted. The Kaplan–Meier method was used to estimate the median OS, PFS, and the 95% CI for each treatment group and subgroup. HRs between treatment groups and their 95% CIs were calculated using the Cox proportional hazards model adjusted by demographic factors selected by a stepwise method for each subgroup. The *P* value of the HR was reported for the descriptive analysis of the difference between the two treatment groups. The *P* value of interaction term is reported for the model with both Tmab+ and Tmab− groups to evaluate association of Tmab use and treatment group. ORR and DCR, and their 95% CIs were estimated for each treatment group. Safety analyses were performed in the safety population (all patients who received at least one dose of study treatment). SAS software (versions 9.3 and 9.4) was used for statistical analyses.

## Results

### Demographics and baseline characteristics

**Fig. 1:**
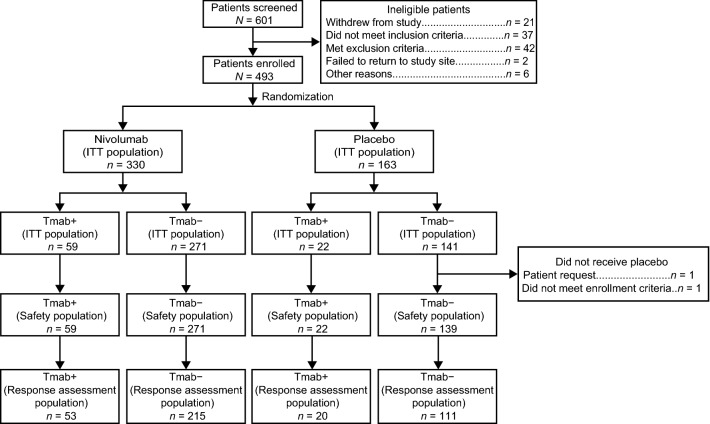
Patient disposition. *ITT* intent to treat, *Tmab* trastuzumab

Overall, 81 patients (nivolumab, *n* = 59; placebo, *n* = 22) had a history of trastuzumab use (Tmab+) and 412 (nivolumab, *n* = 271; placebo, *n* = 141) had no history of trastuzumab use (Tmab−) (Fig. 1). Demographic and baseline characteristics were mostly comparable between patients receiving nivolumab and placebo in both Tmab+ and Tmab− groups. Median age (range) was 62.0 (23.0–83.0) years in patients receiving nivolumab vs 62.5 (33.0–77.0) years in those receiving placebo in the Tmab+ group and 62.0 (20.0–83.0) vs 61.0 (26.0–83.0) years in the Tmab− group, respectively (Table 1). Among the four subgroups, the group of Tmab+ patients receiving nivolumab had a considerably lower proportion of patients with peritoneal metastasis (5/59 [8.5%]). More patients in the Tmab+ groups (nivolumab, 30/59 [50.8%]; placebo, 13/22 [59.1%]) had a history of four or more previous treatment regimens than those in the Tmab− groups (nivolumab, 94/271 [34.7%]; placebo, 59/141 [41.8%]). Notably, previous ramucirumab use was more frequent in the Tmab+ group compared with the Tmab− group (Tmab+: nivolumab, 9/59 [15.3%]; placebo, 4/22 [18.2%] and Tmab−: nivolumab, 26/271 [9.6%]; placebo, 18/141 [12.8%]).

**Table 1:** Patient demographic and baseline characteristics

	Tmab+		Tmab−	
	Nivolumab *n* = 59	Placebo *n* = 22	Nivolumab *n* = 271	Placebo *n* = 141
Sex				
Male	47 (79.7)	20 (90.9)	182 (67.2)	99 (70.2)
Female	12 (20.3)	2 (9.1)	89 (32.8)	42 (29.8)
Age (years), median (range)	62 (23–83)	62.5 (33–77)	62 (20–83)	61 (26–83)
< 65	35 (59.3)	12 (54.5)	154 (56.8)	83 (58.9)
Country				
Japan	31 (52.5)	12 (54.5)	121 (44.6)	62 (44.0)
Korea	25 (42.4)	10 (45.5)	121 (44.6)	64 (45.4)
Taiwan	3 (5.1)	0	29 (10.7)	15 (10.6)
ECOG performance status				
0	19 (32.2)	9 (40.9)	69 (25.5)	38 (27.0)
1	40 (67.8)	13 (59.1)	202 (74.5)	103 (73.0)
Organs with metastases				
< 2	16 (27.1)	3 (13.6)	71 (26.2)	35 (24.8)
≥ 2	43 (72.9)	19 (86.4)	200 (73.8)	106 (75.2)
Site of metastases				
Lymph node	49 (83.1)	18 (81.8)	236 (87.1)	120 (85.1)
Peritoneum	5 (8.5)	8 (36.4)	58 (21.4)	34 (24.1)
Liver	16 (27.1)	4 (18.2)	62 (22.9)	24 (17.0)
Lung	8 (13.6)	1 (4.5)	10 (3.7)	5 (3.5)
Pleura	1 (1.7)	1 (4.5)	3 (1.1)	1 (0.7)
Adrenal	2 (3.4)	1 (4.5)	4 (1.5)	3 (2.1)
Bone	1 (1.7)	2 (9.1)	5 (1.8)	3 (2.1)
Other	6 (10.2)	1 (4.5)	30 (11.1)	16 (11.3)
Number of previous chemotherapy regimens				
2	14 (23.7)	2 (9.1)	55 (20.3)	27 (19.1)
3	15 (25.4)	7 (31.8)	122 (45.0)	55 (39.0)
≥ 4	30 (50.8)	13 (59.1)	94 (34.7)	59 (41.8)
Previous chemotherapy				
Any	59 (100)	22 (100)	271 (100)	141 (100)
Pyrimidine analogs	59 (100)	22 (100)	270 (99.6)	141 (100)
Platinum	59 (100)	22 (100)	252 (93.0)	135 (95.7)
Taxane	53 (89.8)	19 (86.4)	231 (85.2)	121 (85.8)
Irinotecan	42 (71.2)	18 (81.8)	205 (75.6)	105 (74.5)
Ramucirumab	9 (15.3)	4 (18.2)	26 (9.6)	18 (12.8)
Previous gastrectomy				
No	23 (39.0)	8 (36.4)	94 (34.7)	43 (30.5)
Yes	36 (61.0)	14 (63.6)	177 (65.3)	98 (69.5)
Any post-progression therapy				
Radiotherapy	4 (6.8)	0	24 (8.9)	16 (11.3)
Surgery	8 (13.6)	2 (9.1)	59 (21.8)	26 (18.4)
Pharmacotherapy	26 (44.1)	6 (27.3)	102 (37.6)	51 (36.2)
Post-progression pharmacotherapy				
Fluoropyrimidine	12 (20.3)	4 (18.2)	31 (11.4)	20 (14.2)
Taxane	9 (15.3)	2 (9.1)	24 (8.9)	14 (9.9)
Platinum	7 (11.9)	3 (13.6)	23 (8.5)	14 (9.9)
Irinotecan	4 (6.8)	1 (4.5)	10 (3.7)	8 (5.7)
Ramucirumab	10 (16.9)	1 (4.5)	29 (10.7)	11 (7.8)
Immunotherapy	1 (1.7)	0	2 (0.7)	1 (0.7)
Other targeted therapies	1 (1.7)	0	5 (1.8)	5 (3.5)
PD-L1 status (tumor cell) (%)				
≥ 1	2 (6.5)^a^	2 (13.3)^b^	14 (14.1)^c^	8 (17.0)^d^
< 1	29 (93.5)^a^	13 (86.7)^b^	85 (85.9)^c^	39 (83.0)^d^

Data expressed as *n* (%) unless otherwise specified

*ECOG* Eastern Cooperative Oncology Group, *Tmab* trastuzumab, *PD-L1* programmed death-ligand 1

^a^*n* = 31; ^b^*n* = 15; ^c^*n* = 99; ^d^*n* = 47

### Efficacy

**Fig. 2:**
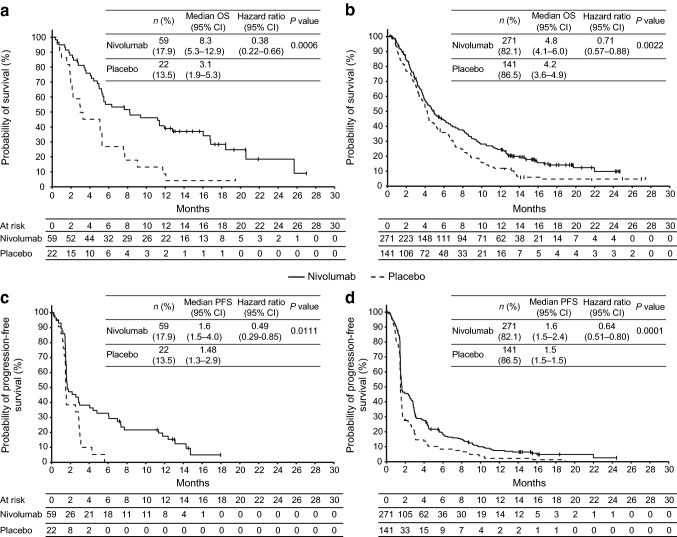
Kaplan–Meier plot of overall survival in **a** Tmab+ and **b** Tmab− patients and progression-free survival in **c** Tmab+ and **d** Tmab− patients. *CI* confidence interval, *ITT* intent to treat, *Tmab* trastuzumab

In both Tmab+ and Tmab− groups, OS was longer in patients receiving nivolumab vs placebo (median [95% CI] OS: Tmab+ group, 8.3 [5.3–12.9] vs 3.1 [1.9–5.3] months, HR [95% CI], 0.38 [0.22–0.66]; *P* = 0.0006 and Tmab−  group, 4.8 [4.1–6.0] vs 4.2 [3.6–4.9] months, HR [95% CI], 0.71 [0.57–0.88]; *P* = 0.0022). The *P* interaction test for the association of trastuzumab use vs nivolumab with OS was significant (*P* = 0.0431) (Table 2). Survival curves for OS showed a consistent advantage with nivolumab vs placebo in both Tmab +  and Tmab−  patients (Fig. 2a, b).

**Table 2:** Efficacy analysis of nivolumab by previous use of trastuzumab

	Tmab+		Tmab−	
	Nivolumab *n* = 59	Placebo *n* = 22	Nivolumab *n* = 271	Placebo *n* = 141
BOR, *n* (%)				
PR	10 (16.9)	0	21 (7.7)	0
SD	15 (25.4)	7 (31.8)	62 (22.9)	26 (18.4)
PD	23 (39.0)	10 (45.5)	101 (37.3)	69 (48.9)
NE	11 (18.6)	5 (22.7)	87 (32.1)	44 (31.2)
ORR, *n* (%)^a^	10 (16.9)	0	21 (7.7)	0
DCR, *n* (%)^a^	25 (42.4)	7 (31.8)	83 (30.6)	26 (18.4)
OS (months), median (95% CI)	8.3 (5.3–12.9)	3.1 (1.9–5.3)	4.8 (4.1–6.0)	4.2 (3.6–4.9)
HR (95% CI)^a^	0.38 (0.22–0.66)		0.71 (0.57–0.88)	
*P* value	0.0006		0.0022	
P interaction, P value^b^	0.0431			
PFS (months), median (95% CI)	1.6 (1.5–4.0)	1.5 (1.3–2.9)	1.6 (1.5–2.4)	1.5 (1.5–1.5)
HR (95% CI)^c^	0.49 (0.29–0.85)		0.64 (0.51–0.80)	
*P* value	0.0111		0.0001	
P interaction, P value^b^	0.3046			
DOR (months), median (range)	8.6 (4.3–13.1)	–	9.5 (2.8–22.9)	–
TTR (months), median (range)	3.0 (1.4–7.0)	–	1.6 (1.4–6.2)	–

*BOR* best overall response, *CI* confidence interval, *DCR* disease control rate, *DOR* duration of response, *HR* hazard ratio, *ORR* objective response rate, *OS* overall survival, *PD* progressive disease, *PFS* progression-free survival, *PR* partial response, *SD* stable disease, *Tmab* trastuzumab, *TTR* time to response

^a^Adjustment factors: Tmab+, post-progression therapy surgery; Tmab−, organs with metastases (< 2), age (< 65 years), and recurrence site (peritoneum)

^b^*P* interaction represents the association of Tmab use vs nivolumab with OS or PFS

^c^Adjustment factors: Tmab+, organs with metastases (< 2); Tmab−, age (< 65 years) and recurrence site (liver)

Benefit of nivolumab for PFS was also comparable in both Tmab +  and Tmab−  groups (median [95% CI] PFS: Tmab +  group, 1.6 [1.5–4.0] vs 1.5 [1.3–2.9] months, HR [95% CI], 0.49 [0.29–0.85]; *P* = 0.0111 and Tmab−  group, 1.6 [1.5–2.4] vs 1.5 [1.5–1.5 months], HR [95% CI], 0.64 [0.51–0.80]; *P* = 0.0001). The *P* interaction test for the association of trastuzumab use vs nivolumab with PFS was not significant (*P* = 0.3046) (Table 2). Notably, median PFS with nivolumab was similar in the Tmab+ and Tmab− groups. Kaplan–Meier curves for PFS of nivolumab and placebo initially overlapped in both Tmab+ and Tmab− groups but separated later (Fig. 2c, d).

ORR of patients receiving nivolumab was 16.9% in the Tmab+ group and 7.7% in the Tmab− group. None of the patients receiving placebo in either group showed any response (Table 2). A higher DCR was observed in patients receiving nivolumab than in those receiving placebo, irrespective of Tmab status (Tmab+, 42.4% vs 31.8%; Tmab−, 30.6% vs 18.4%) (Table 2). Median (range) TTR was 3.0 (1.4–7.0) months and 1.6 (1.4–6.2) months in the Tmab+ and Tmab− groups receiving nivolumab, respectively (Table 2). Median (range) DOR was 8.6 (4.3–13.1) months and 9.5 (2.8–22.9) months in the Tmab+ and Tmab− groups receiving nivolumab, respectively.

The proportion of patients showing some tumor shrinkage (> 1%) was higher in the nivolumab group than in the placebo group in both Tmab+ and Tmab− groups (Tmab+, 38.8% vs 16.7%; Tmab−, 36.4% vs 10.5%).

### Safety

Overall, incidence of any AE in patients receiving nivolumab was comparable between the Tmab+ and Tmab− groups (54 [91.5%] vs 247 [91.1%]) (Online resource 1: Table). Incidence of any treatment-related AE in patients receiving nivolumab was slightly higher in the Tmab+ group than in the Tmab− group (31 [52.5%] vs 111 [41.0%]), whereas the incidence of treatment-related serious AEs was comparable (6 [10.2%] vs 29 [10.7%]). The most frequent (≥ 10% in any group) treatment-related AEs among patients receiving nivolumab in the Tmab+ and Tmab− groups were pruritus (15.3% and 7.7%, respectively) and rash (13.6% and 4.8%, respectively) (Table 3). Treatment-related AEs of special interest (frequency ≥ 2% in any group) in the Tmab+ and Tmab− groups included interstitial lung disease (3.4% and 1.5%, respectively) and maculopapular rash (5.1% and 0%, respectively). Among the patients receiving nivolumab, treatment-related AEs leading to discontinuation of treatment were reported in 3 (5.1%) and 6 (2.2%) patients, and those leading to delayed dosing were reported in 7 (11.9%) and 22 (8.1%) patients in the Tmab+ and Tmab− groups, respectively. Treatment-related death was reported in one (1.7%) and eight (3.0%) patients in the Tmab+ and Tmab− groups receiving nivolumab, respectively.

**Table 3:** Safety analysis of nivolumab by previous use of trastuzumab

	Tmab+		Tmab−	
	Nivolumab *n* = 59	Placebo *n* = 22	Nivolumab *n* = 271	Placebo *n* = 139
Common treatment-related AEs^a^				
Pruritus	9 (15.3)	3 (13.6)	21 (7.7)	6 (4.3)
Rash	8 (13.6)	1 (4.5)	13 (4.8)	4 (2.9)
Diarrhea	4 (6.8)	0	19 (7.0)	3 (2.2)
Malaise	3 (5.1)	0	10 (3.7)	6 (4.3)
Nausea	3 (5.1)	0	12 (4.4)	4 (2.9)
ALT increased	1 (1.7)	0	7 (2.6)	1 (0.7)
AST increased	1 (1.7)	1 (4.5)	10 (3.7)	2 (1.4)
Fatigue	1 (1.7)	2 (9.1)	17 (6.3)	7 (5.0)
Hypothyroidism	1 (1.7)	0	10 (3.7)	1 (0.7)
Decreased appetite	0	0	16 (5.9)	7 (5.0)
Pyrexia	0	0	9 (3.3)	3 (2.2)
Treatment-related AEs of special interest				
Maculopapular rash	3 (5.1)	0	0	0
Interstitial lung disease	2 (3.4)	0	4 (1.5)	0
Hypopituitarism	1 (1.7)	0	0	0
Hypothyroidism	1 (1.7)	0	10 (3.7)	1 (0.7)
Acute hepatitis	0	0	1 (0.4)	0
Autoimmune thyroiditis	0	0	1 (0.4)	0
Colitis	0	0	2 (0.7)	0
Hyperthyroidism	0	0	2 (0.7)	0
Pneumonitis	0	0	1 (0.4)	0

All data presented as *n* (%)

*AE* adverse event, *ALT* alanine aminotransferase, *AST* aspartate aminotransferase, *Tmab* trastuzumab

^a^Events that occurred in ≥ 2% of patients receiving nivolumab in the Tmab+ or Tmab− group

## Discussion

In this post hoc analysis, nivolumab improved OS, PFS, ORR, DCR, and reduction in tumor burden compared with placebo in both Tmab+ and Tmab− groups. Patients treated with nivolumab had a sustained and durable response compared with placebo in both Tmab+ and Tmab− groups. Safety of nivolumab was comparable between Tmab+ and Tmab− patients.

Trastuzumab in combination with platinum-based chemotherapy is the standard first-line treatment in HER2+ gastric cancer patients [2-4] based on results from ToGA [7]. Although clinical trials using new anti-HER2 agents were successful for metastatic breast cancer, the results from previous studies conducted in the first- or second-line setting for HER2+ gastric cancer patients, however, were disappointing. As the first-line palliative chemotherapy treatment in the phase 3 trial (TRIO-013/LOGiC), patients with HER2+ advanced G/GEJ cancer received lapatinib (an anti-HER2 agent) or placebo in combination with capecitabine plus oxaliplatin (CapeOX); OS was not significantly different (median OS, 12.2 vs 10.5 months; HR, 0.91 [95% CI 0.73–1.12]; *P* = 0.3492) [20]. Moreover, in a phase 3 trial (JACOB), metastatic G/GEJ cancer patients received pertuzumab (another anti-HER2 antibody) or placebo in combination with trastuzumab plus chemotherapy (standard cisplatin/fluoropyrimidine regimen) as first-line treatment; OS was not significantly different between the pertuzumab and placebo arms (median OS, 17.5 vs 14.2 months; HR, 0.84 [95% CI 0.71–1.00]; *P* = 0.0565) [21]. As the second-line treatment in a randomized phase 2 study conducted by the West Japan Oncology Group (WJOG7112G [T-ACT]), trastuzumab plus paclitaxel showed no benefit over paclitaxel alone in patients with HER2+ advanced G/GEJ cancer refractory to first-line trastuzumab plus chemotherapy [8]. Development of new active agents for HER2+ G/GEJ cancer is warranted.

Nivolumab is recommended as a third-line or later-line therapy in gastric cancer patients who are likely to have received trastuzumab. Previous reports are suggestive of the impact of anti-HER2 therapy on the expression of PD-L1 [15-18] and of the decrease in HER2 expression in some patients during treatment with trastuzumab [22, 23]. It was thus hypothesized that HER2 expression and/or prior use of trastuzumab might have had some influence on the efficacy of nivolumab in the ATTRACTION-2 trial. In this analysis, nivolumab showed similar efficacy regardless of prior trastuzumab use. However, the HR for OS was better in the Tmab+ group than in the Tmab− group (interaction *P* = 0.0431). Apart from prior trastuzumab use, the proportion of patients receiving post-progression pharmacotherapy was slightly higher in the Tmab+ group treated with nivolumab. Because OS is affected by many other factors, including the patient’s background and post-progression pharmacotherapy, the efficacy of nivolumab is best represented by the response rate and PFS. In terms of HR for PFS, no difference was observed between the Tmab+ and Tmab− groups (interaction *P* = 0.3046). Therefore, it could not be deduced that nivolumab showed better efficacy in HER2+ G/GEJ cancer patients. To say the least, the results of this study suggest that nivolumab can be efficacious regardless of HER2 expression status.

Also, considering the patient background, fewer patients receiving nivolumab had peritoneal metastases in the Tmab+ than in the Tmab− group (8.5% vs 21.4%, respectively). On the other hand, the proportion of patients receiving nivolumab who had received ≥ 4 previous treatment regimens was higher in the Tmab+ group compared with the Tmab− group (50.8% vs 34.7%, respectively). Furthermore, 15.3% and 9.6% of patients receiving nivolumab in the Tmab+ and Tmab− groups, respectively, had been previously treated with ramucirumab. These differences in prior chemotherapy suggest that fewer active agents were available after nivolumab for the Tmab+ group than the Tmab− group. Therefore, it is difficult to explain the longer OS in the Tmab+ group based on patient background alone.

As the relationship between HER2 and PD-L1 expression is still unknown, further investigation will be required to examine whether nivolumab provides longer survival benefit in HER2+ G/GEJ cancer and to elucidate the mechanisms involved.

The safety profile of nivolumab both in Tmab+ and Tmab− patients with advanced G/GEJ cancer was similar to that previously reported and known for nivolumab. Most AEs and treatment-related AEs were mild to moderate in severity; no unexpected AEs were observed.

There are certain limitations associated with this study. The number of HER2+ patients was small and associated with substantial differences in patient backgrounds. The HER2 status of patients immediately before enrollment and its change during treatment was not assessed. In addition, PD-L1 status was assessed using archival tissues before trastuzumab use in a limited number of patients. Therefore, it is not clear whether trastuzumab treatment may have altered PD-L1 expression, just as lapatinib and afatinib have previously been shown to suppress PD-L1 expression via the EGFR/HER2 signaling blockade [17]. Although the higher proportion of patients receiving post-progression pharmacotherapy is one of the factors that could have contributed to the significantly longer OS in the Tmab+ group between nivolumab and placebo, we did not evaluate its influence on the OS.

## Conclusions

The efficacy and safety of nivolumab for advanced G/GEJ cancer were consistent regardless of prior trastuzumab use. We anticipate the future development of anti-PD-1 agents including nivolumab for HER2+, advanced G/GEJ cancer.
